# Four Weeks of Treatment With Rifaximin Fails to Significantly Alter Microbial Diversity in Rectal Samples of HIV-Infected Immune Non-Responders (ACTG A5286) Which May be Attributed to Rectal Swab Use

**DOI:** 10.20411/pai.v4i2.290

**Published:** 2019-09-23

**Authors:** Brett B. Williams, Stefan J. Green, Ronald J. Bosch, Ellen S. Chan, Jeffrey M. Jacobson, David M. Margolis, Phillip Engen, Alan L. Landay, Cara C. Wilson, A5286 protocol team

**Affiliations:** 1 Division of Infectious Disease; Rush University Medical Center; Chicago, Illinois; 2 Sequencing Core; University of Illinois at Chicago; Chicago, Illinois; 3 Center for Biostatistics in AIDS Research; Harvard School of Public Health; Boston, Massachusetts; 4 Division of Infectious Diseases and HIV; Drexel University; Philadelphia, Pennsylvania; 5 Department of Medicine; University of North Carolina; Chapel Hill, North Carolina; 6 Division of Gastroenterology, Hepatology and Nutrition; Department of Medicine; Rush University Medical Center; Chicago, Illinois; 7 Department of Immunology and Microbiology; Rush University Medical Center; Chicago, Illinois; 8 Department of Medicine; University of Colorado at Denver; Aurora, Colorado

**Keywords:** HIV, microbiome, immune activation, microbial translocation, rifaximin

## Abstract

**Introduction:**

HIV-infected individuals have evidence of intestinal microbial translocation which is associated with immune activation and unfavorable clinical outcomes. Rifaximin, a non-absorbable antibiotic which reduces microbial translocation in other disease states, was shown to have a marginal beneficial effect on microbial translocation, T-cell activation, and inflammation in a multisite randomized trial (ACTG A5286; NCT01466595) of HIV-infected persons with poor immunologic recovery receiving ART. Here, we report analysis of the rectal microbiome changes associated with that trial.

**Methods:**

HIV-1-infected individuals receiving ART with CD4-T cell count < 350cells/mm^3^ and viral suppression were randomized 2:1 to rifaximin or no therapy for 4 weeks. Rectal swabs were collected at baseline (pre-treatment) and at week 4 of rifaximin therapy. Genomic DNA extracted from rectal swab samples was analyzed using high throughput sequencing and quantitative PCR of bacterial 16S ribosomal RNA (rRNA) genes.

**Results:**

Forty-eight HIV-infected participants (31 received rifaximin, 17 no treatment) were included. There was broad variability in the recovery of bacterial rRNA from the specimens at baseline. No major significant (FDR *P* < 0.05) effects of rifaximin treatment on alpha- or beta-diversity or individual taxa were observed between or within the treatment arms, with analyses conducted at taxonomic levels from phylum to genus.

**Conclusions:**

Rifaximin did not meaningfully alter the diversity or composition of the rectal microbiome of HIV-infected individuals after 4 weeks of therapy, although rectal swab specimens varied widely in their microbial load.

## INTRODUCTION

The collection of microorganisms that colonize the gastrointestinal tract of humans, or the gut microbiome, influences many different inflammatory disease processes [1, 2]. In particular, persons living with HIV infection have been reported to have a gut microbiome containing more pro-inflammatory bacteria than seronegative individuals [3-5]. It is hypothesized that this shift in the microbiome, along with other HIV-associated changes in the intestinal mucosa and gut barrier, contributes to the increased microbial translocation and concomitant systemic inflammation and immune activation that characterize all stages of HIV disease [3-5]. Several groups, including the AIDS Clinical Trials Group (ACTG), have demonstrated a link between excess microbial translocation, persistent immune activation, and morbidity and mortality in persons living with HIV[6-8]. Individuals with inadequate peripheral blood CD4+ T-cell recovery (< 350cells/mm^3^) following viral suppression have a particularly notable disrupted gut barrier (zonulin-1, I-FABP) and immune activation as measured by soluble CD14 (sCD14), IL-6 and LPS, which may be due in part to limited intestinal CD4+ T-cell recovery [9-11].

Rifaximin, an oral, non-absorbed antibiotic, reduces circulating lipopolysaccharide (LPS) levels and improves hepatic encephalopathy in persons with cirrhosis [12]. In ACTG A5286, we tested the hypothesis that 4 weeks of rifaximin reduces markers of microbial translocation (including LPS and soluble CD14) and immune activation in HIV-positive individuals with poor CD4 recovery despite full virologic suppression [13]. In that study, rifaximin administration only marginally decreased markers of T-cell activation, microbial translocation, and systemic inflammation. In this secondary analysis, we evaluated whether rifaximin altered the composition or diversity of gut resident microbial communities in a subset of A5286 participants with available paired rectal swabs.

## METHODS

### Study Population

ACTG A5286 was a 4-week, randomized, open-label study in which 65 eligible individuals who were HIV positive, with CD4 < 350c/mm^3^, with plasma HIV RNA below the limit of detection, and receiving ART for at least 48 consecutive weeks were randomized to receive rifaximin 550 mg by mouth 2 times daily for 4 weeks or no treatment. The full study inclusion and exclusion criteria and the primary results have been previously reported [13]. Institutional review board approval was obtained by each ACTG site. Participants provided written informed consent.

The effect of rifaximin on the gut microbiome was evaluated in available pre-treatment (baseline) and post-treatment (week 4) anal swab samples selected from the primary analysis population of ACTG A5286.

### Rectal Swab Collection

Rectal swabs were inserted into the anal canal, beyond the anal verge (±3 cm), with five circular motions in performing the collection. Then, the rectal swabs were snap frozen in liquid nitrogen and stored at -80°C until use.

### Characterization of Microbial Community Composition

Genomic DNA was extracted from rectal swab samples using the FastDNA Spin Kit for Soil, (MP Biomedicals, Solon, OH), according to the manufacturer's instructions. Quantification of bacterial small subunit rRNA (SSU or 16S rRNA) gene abundance was performed as described previously using Taqman 2× Gene Expression Master Mix (Invitrogen, Foster City, CA) [14]. Primers and probe were ordered from Integrated DNA Technologies (Coralville, IA). Absolute quantification was performed using a standard curve derived from PCR products generated by near-full gene amplification of 16S rRNA genes using the general bacterial primer set 27F and 1492R. The standard curve was linear across a scale of 7 orders of magnitude (from 6.85E+01 to 6.85E+08 copies/reaction), with an efficiency of 84%. Assays were performed in triplicate in 384-well plates in a volume of 10 uL per sample, using a ViiA7 real-time PCR instrument (Life Technologies). All qPCR assays were performed at the University of Illinois at Chicago Sequencing Core (UICSQC).

Fecal microbiomes were characterized using deep sequencing of PCR-amplified portions of microbial 16S rRNA genes. Sequencing was performed at Argonne National Laboratory, using the standard Earth Microbiome Project primers (ie, 515F/806R) containing sample-specific barcode sequences and Illumina adapter sequences [15]. Amplicons were pooled and sequenced on an Illumina MiSeq sequencer, implementing V2 chemistry with paired-end 2x150 base reads.

Forward and reverse reads were merged and were quality trimmed, and sequences longer than 200 bases were exported (v7.0, CLC Bio, Qiagen, Boston, MA). Sequences were screened for chimeras (usearch61 algorithm), and putative chimeric sequences were removed from the dataset (QIIME v1.8.0) [16, 17]. Each sample sequence set was sub-sampled (rarefied) to 6,500 sequences and data were pooled, renamed, and clustered into operational taxonomic units (OTU) at 99% similarity (usearch61algorithm) [18]. A depth of 6,500 sequences was used as this number represented approximately 75% of the sequences from the sample with the fewest sequences. Representative sequences from each OTU were extracted and classified using the uclust consensus taxonomy assigner (Greengenes 13_8 reference database). A biological observation matrix (BIOM; a taxon-by-sample abundance table) was generated at each taxonomic level (“make OTU table” algorithm) and analyzed and visualized using the software package Primer 6 [19-21]. To compare between samples, the Bray-Curtis metric was used, as implemented within Primer 6. Comparisons between different treatment groups and sampling visits were performed using analysis of similarity (ANOSIM) at various taxonomic levels. Data were standardized within samples and then square-root transformed. Differences in the relative abundance of individual taxa between a priori defined treatment or visit groups were detected using a Kruskal-Wallis test generating a Benjamini-Hochberg false-discovery rate (FDR) corrected *P*-value. The FDR adjustment is used to address type I errors (false positives) when dealing with a large number of variables in a single analysis. For microbial community analyses, analyses are performed to detect significant differences in the relative abundance of many taxa, and in these studies, approaches such as the Bonferroni correction have been too stringent [22]. Taxa with an average abundance of < 1% across the sample set were removed from these analyses.

Alpha diversity indices, which represent within-sample diversity measures (eg, number of taxa present, evenness of relative abundance of taxa, and hybrid measures of both–such as the Shannon Index) were calculated using the software package Primer 6. Analyses were performed at the taxonomic level of genus, as the short-read amplicon sequence data (ie, 252-253 bases in length for each sequence) are not sufficiently long for consistent annotation at the species level. Most sequences could be annotated at the taxonomic level of genus, and this taxonomic level was therefore used for comparative analyses of diversity. Differences in the alpha diversity indices between groups were tested for significance using the Mann-Whitney non-parametric U-test implemented within the software package Origin (Origin2015). Furthermore, comparison of between-visit microbial community structure was performed using the Bray-Curtis metric, and the distribution of baseline vs week 4 similarity within participants from the Rifaximin and the no-treatment arms were measured. A Mann-Whitney test was performed to determine if the shift in community from baseline to week 4 was significantly different between study arms. Stacked histograms were generated for visualization purposes in Origin.

A series of ANOSIM calculations were performed at multiple taxonomic levels to determine if microbial community structure was significantly different between study arms, and between visits within study arms. In addition, group-significance (Kruskal-Wallis) test results, run at all taxonomic levels from phylum to species, were compiled. Significance was defined as FDR *P* < 0.05.

### Statistical Analysis of 16S rRNA Gene Abundance Measured by qPCR

Analysis was an as-treated analysis that was limited to participants in both arms who had data for baseline and week 4, and (for the rifaximin arm) who remained on study treatment through week 4 (allowing ≤ 6 missed doses). These participants did not change ART, use prohibited medications, or have virologic failure during this time period. All statistical tests were 2-sided at the 0.05 nominal level of significance without adjustments for multiple testing.

## RESULTS

Participants in A5286 who provided rectal swab samples from both visits and from whose samples at least 6,500 sequences were generated, were included in the analysis. After removing participants whose samples did not meet these criteria, 48 participants (31 from the rifaximin arm; 17 from the no-treatment arm) had complete data for this analysis (Table 1), yielding a total of 96 samples for analysis. The rifaximin and no-treatment groups were similar in baseline characteristics. Overall, the median age was 50 (years), 90% male, 52% white non-Hispanic, 31% black non-Hispanic, and 15% Hispanic. The median CD4 count was 229 and duration of undetectable viral load was 3.3 years.

Quantitative analysis of bacterial 16S rRNA gene abundance was performed using quantitative PCR to determine if rifaximin treatment resulted in a shift in the abundance of bacteria in each sample. Total bacterial 16S rRNA gene abundance was not significantly different following treatment with rifaximin, although the absolute abundance in samples varied across 7 orders of magnitude (Figure 1A). The variability between time points was similar in the rifaximin and no-treatment groups; it is possible that small variations due to rifaximin may have been obscured by the relatively high variation associated with rectal swabs as a collection method.

**Figure 1A.**
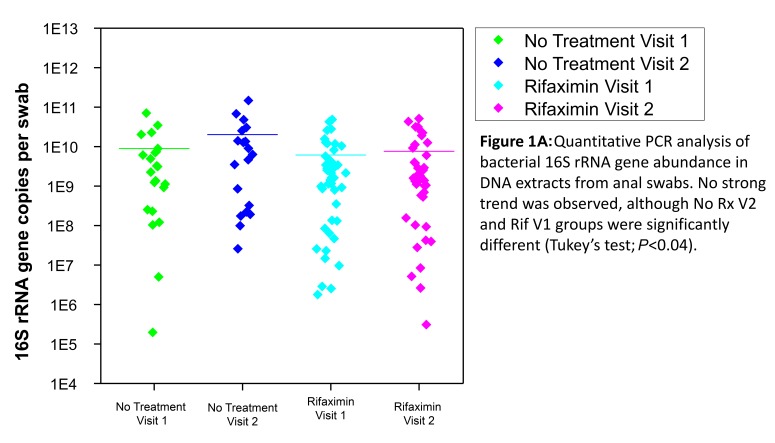
Quantitative PCR analysis of bacterial 16S rRNA gene abundance in DNA extracts from anal swabs. No strong trend was observed, although no-treatment visit 2 and Rifaximin visit 1 groups were significantly different (Tukey's test; *P* < 0.04).

To determine if microbial community composition between visits within each individual was more variable in the no-treatment or rifaximin arms, Bray-Curtis similarity values for each individual (baseline vs week 4) were calculated. No significant difference was observed for the within-group comparison of these values, suggesting that rifaximin did not alter the variability of the microbial community composition between visits.

Similarly, we assessed microbial community diversity to determine if there were significant changes in alpha diversity indices between treatment arms and between visits within treatment arms. An analysis of the Shannon index (a combined measure of taxon richness and evenness) between groups did not reveal a systematic shift in diversity between treatments or visits (Figure 1B). A significant difference in Shannon index (taxonomic level of genus) was observed between control and rifaximin arm samples at visit 2 (4 weeks), which appears to have been driven by a trend toward increased alpha diversity in the rifaximin arm at baseline. No difference was seen at the species level. In each of the groups, sequences from bacteria of the phylum Firmicutes predominated (group average relative abundance ranged from 53.3%-62.5%), followed by Bacteroidetes (22.3%-27.6%) and Proteobacteria (5.6%-10.5%) (Figure 1c). The genus *Prevotella* was the most prevalent genus (11.8%-16.7%) in all groups at all time points.

**Figure 1B.**
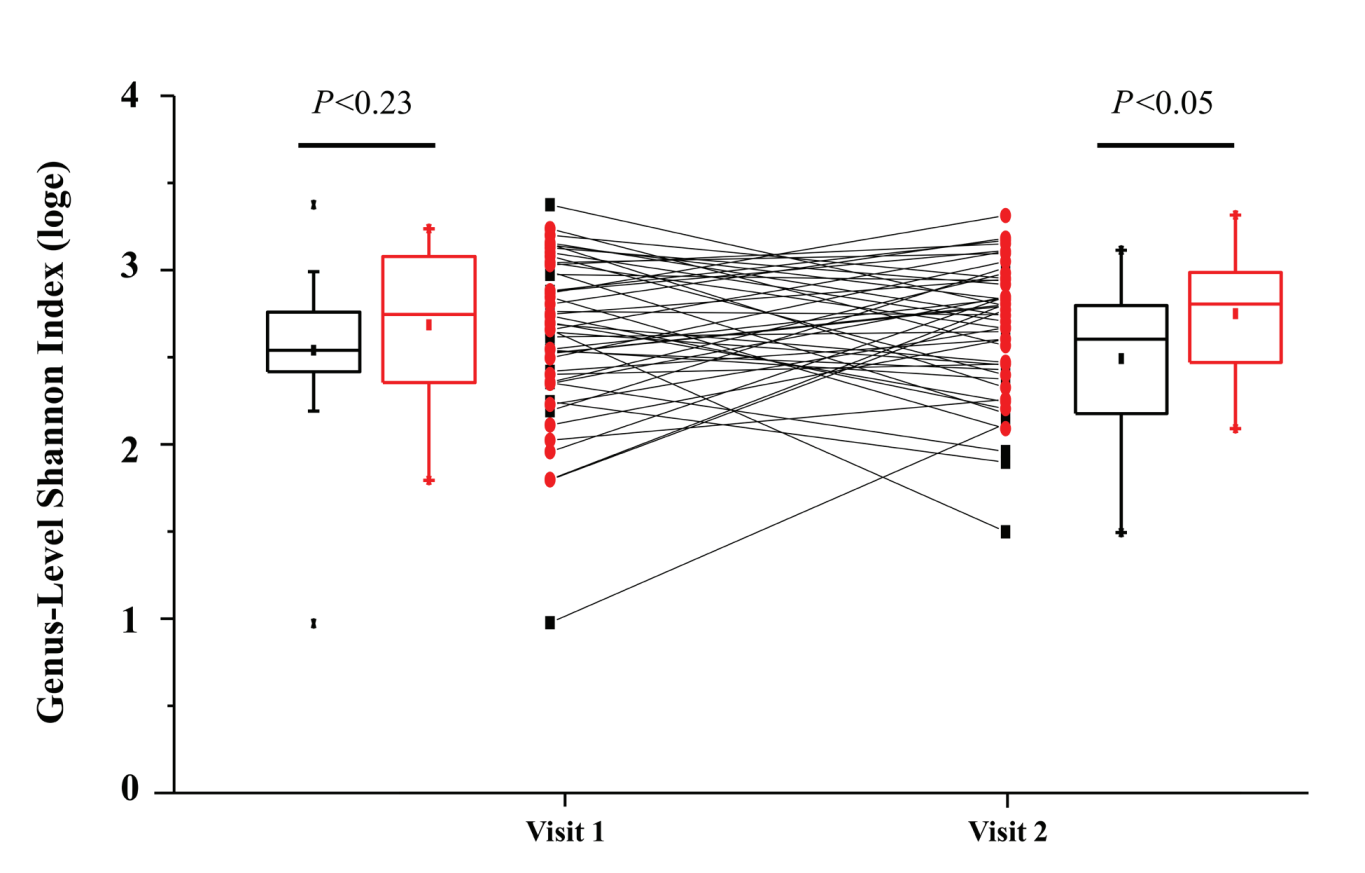
Change in fecal microbial diversity between visit 1 (before treatment) and visit 2 (after treatment) for individuals taking rifaximin (red circles) and for individuals taking no study treatment (black squares). No significant difference in Shannon Index between treatment groups was observed at baseline (visit 1; Mann-Whitney U-test), but a significant difference was observed at visit 2.

**Figure 1C.**
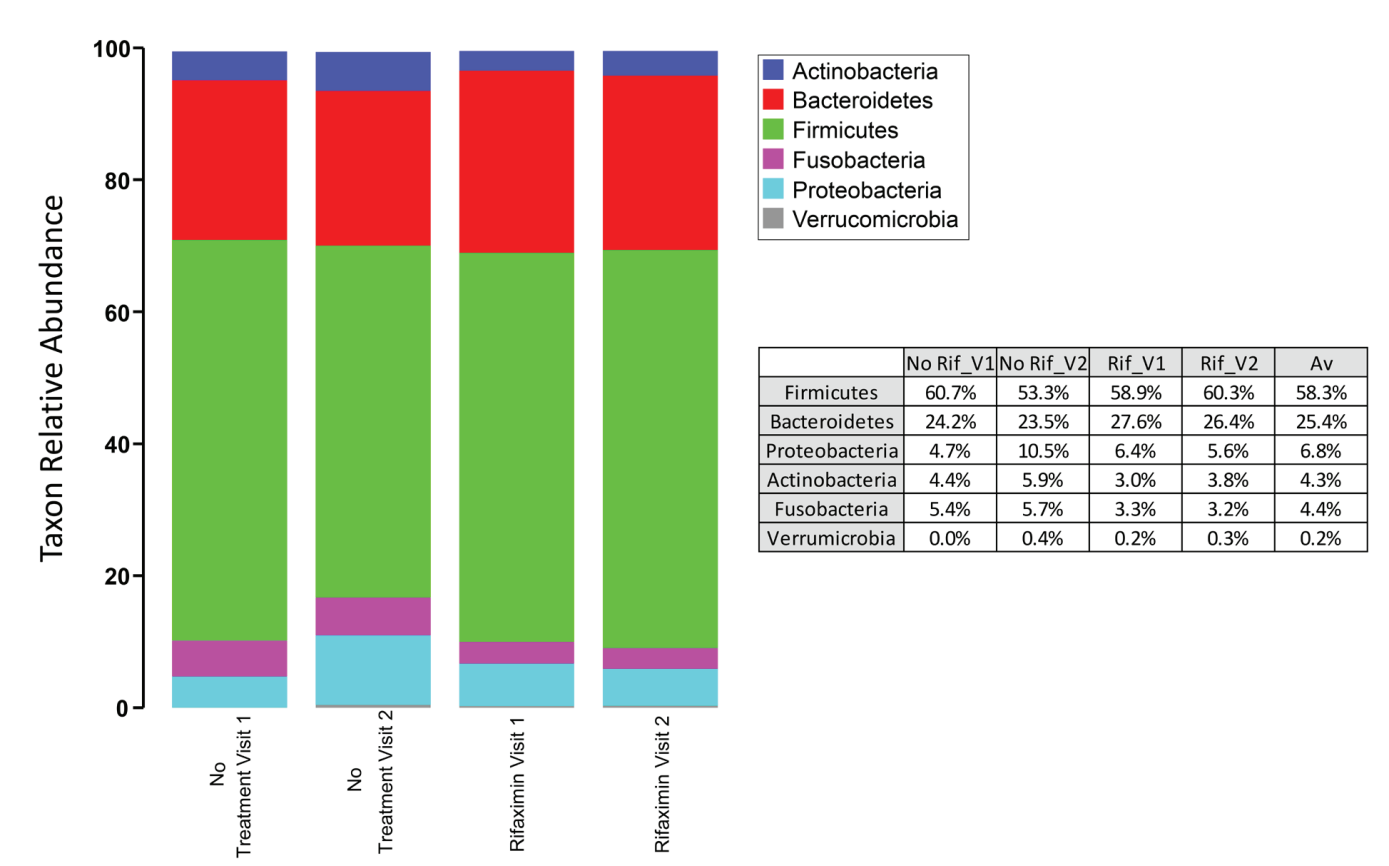
Phylum-level microbial community composition of fecal microbiome samples from individuals in the rifaximin and placebo experimental arms. Average abundance of each taxon is shown among 31 individuals (rifaximin arm) and 17 individuals (no-treatment arm). Each sample was rarefied to 6500 sequences and average values for each taxon are shown. No taxa are significantly different between control and rifaximin arms at visit 2. Greater than 99% of all sequences belonged to 1 of the 6 phyla shown.

No significant difference in microbial community structure within treatment arms between baseline and week 4 was observed (ANOSIM global R values -0.022 to -0.029; *P*-values 0.811-0.925). At the second visit, the microbial communities of the no-treatment control and rifaximin arms were significantly different, (ANOSIM global R = 0.099; *P*-value = 0.041) and this was similar to what was seen with the Shannon diversity index. At both baseline and week 4, there were no differences in the phylum or species level assessment, and only 1 genus level difference was identified between the 2 groups: the genus *Sutterella* was more abundant in the rifaximin arm at baseline (FDR value < 0.05). The average relative abundance of sequences for each group annotated as *Sutterella* ranged from 0.3% to 1.4%. However, no individual taxa were significantly different between the 2 treatment arms at week 4 (FDR *P*-value < 0.05).

## DISCUSSION

After 4 weeks of rifaximin therapy, gut microbiome diversity and composition did not change significantly in a cohort of HIV-infected persons with suppressed HIV viremia and CD4 counts below 350 cells/mm^3^. At week 4, there was a modest difference in genus level diversity as measured by the Shannon index between the rifaximin and the no-treatment groups although this difference likely reflects differences present at baseline. The difference in the genus *Sutterella* at baseline which disappeared following treatment is unlikely to be meaningful as it was not associated with any other major changes in the microbiome over time. Lack of substantial change in rectal microbiota between baseline and week 4 in the rifaximin group may explain the marginal effect of rifaximin therapy on markers of microbial translocation and immune activation seen in the parent study [13]. The parent study was designed based on findings that endotoxemia was driving persistent immune activation and that rifaximin therapy resulted in reductions in endotoxemia in participants with hepatic encephalopathy, however these findings were based on 8 weeks of rifaximin therapy [23]. In the hepatic encephalopathy study mentioned above, no major changes were noted in the microbial abundance after 8 weeks of therapy, but alterations in gut bacterial linkages to metabolites were noted [23]. Previous studies also showed that antibiotic therapy reduced serum LPS levels, a marker of microbial translocation, in SIV-infected nonhuman primates [6]. More recently, combination therapy with rifaximin and sulfasalazine was demonstrated to reduce microbial translocation and immune activation as well as prevent mucosal CD4 depletion in acute SIV infection [24]. Conversely, no significant effect on microbial translocation or immune activation was noted when this combination was given to chronically SIV-infected animals, consistent with findings from the A5286 parent study which included chronically infected humans [13, 24]. Thus, an alternative explanation is that in chronic infection, and particularly in persons with advanced disease (ie, low nadir CD4 count), rifaximin is not an effective intervention as the changes to gut mucosal integrity and immune surveillance are beyond repair.

While Pandrea et al did not measure alterations in the fecal microbiota in their main study, they did demonstrate, using a sub-population, that 2 weeks of rifaximin treatment of SIV-naive animals resulted in changes in the fecal microbiota composition of stool, a significant reduction in fecal biodiversity, and reduced fecal bacterial loads [24]. Specifically, changes in fecal microbiome composition were dominated by an increase in the abundance of Prevotellaceae [24]. The inability of rifaximin to reduce inflammation during chronic infection may be related to severe pre-existing mucosal immune disruption and a resultant dysbiosis, associated with an increase in Prevotellaceae, which may only be detectable in stool or more proximal samples [3, 4, 25, 26]. However, one large cohort study suggested that the increase in Prevotellaceae may be an enterotype found in men who have sex with men (MSM) rather than being related to HIV infection [27].

This study had several limitations. First, our sample size was relatively small, although on par with several prior HIV specific microbiome studies [3-5, 26, 28-30]. The intervention was of relatively short duration and any change that occurred after the week 4 visit would have been missed. The microbiome analysis was conducted at 4 weeks while the primary published analysis, where the authors showed that the use of rifaximin led to a reduction in endotoxemia, was based on an 8-week analysis. It would have been more appropriate to analyze the changes in the microbiome at the same time point, but samples are not available from the 8-week time point.

Data on potentially confounding factors such as diet, anal sex, and tobacco or alcohol intake were not collected, though the effect of these factors should have been mitigated by randomization. Two of our findings suggest that the particular method of obtaining rectal swabs used for this study may result in too much variability. Bray-Curtis similarity comparing baseline to the week 4 microbiome (Figure 1B) was quite low (< 40%) in the no-treatment group, suggesting poor within-subject reproducibility over time compared to prior publications, and the bacterial load per swab varied by approximately 4 logs between samples. These findings suggest that swab-to-swab variability was high and may be dependent on factors such as time between last bowel movement and swab collection technique. Because this was a multi-site study, all rectal swabs were not collected by the same individual and there was likely variability in technique. The high swab-to-swab variability likely reduced our power to measure any small changes caused by rifaximin. Single-center studies have found rectal swab microbiota sampling to be fairly reliable and to correlate well with more proximal gut samples [31, 32]. Future studies should evaluate the validity and reproducibility of rectal swab specimens for intestinal microbiome analysis using a comparison between rectal swab, stool, and sigmoid biopsy samples in both healthy participants and those with pathology.

We failed to identify any effects of 4 weeks of rifaximin on the rectal microbiome. Our results appear to confirm those of groups investigating rifaximin in human cohorts with hepatic encephalopathy, irritable bowel syndrome, or inflammatory bowel disease (IBD) which found minimal or no change in the microbiome following rifaximin therapy [21, 33, 34]. The change in microbiota induced by rifaximin may be temporary as at least some microbes can quickly develop resistance to rifaximin; thus, we may have missed this change by sampling only after 4 weeks of treatment [35]. For example, Pandrea et al noted a change at 2 weeks in their SIV model [24]. Sub-inhibitory levels of rifaximin have been demonstrated to downregulate virulence factors and the IL-8 induction potential of gastrointestinal pathogenic Enterobacteriaceae [36]. This may suggest that gut bacteria may rapidly adapt to survive the presence of rifaximin, yet continue to have altered expression of virulence factors, which could explain the therapeutic effects seen in hepatic encephalopathy and IBD. The symptomatic improvement seen in traveler's diarrhea with rifaximin despite failure to eradicate the causative strain of Enterobacteriaceae also suggests that rifaximin may be better at downregulating virulence factors than it is at killing [37, 38].

## CONCLUSIONS

Rifaximin did not significantly alter the composition or diversity of the colon microbiome of HIV-infected individuals as assessed by rectal swabs, although specimens varied widely in their microbial load and composition. Future studies of the colon microbiome should compare rectal swab samples to more proximal samples. Further investigation of microbial metabolites and virulence factors may be helpful in elucidating the mechanisms by which rifaximin reduces microbial translocation in other disease states.

## Figures and Tables

**Supplementary Figure 1.**
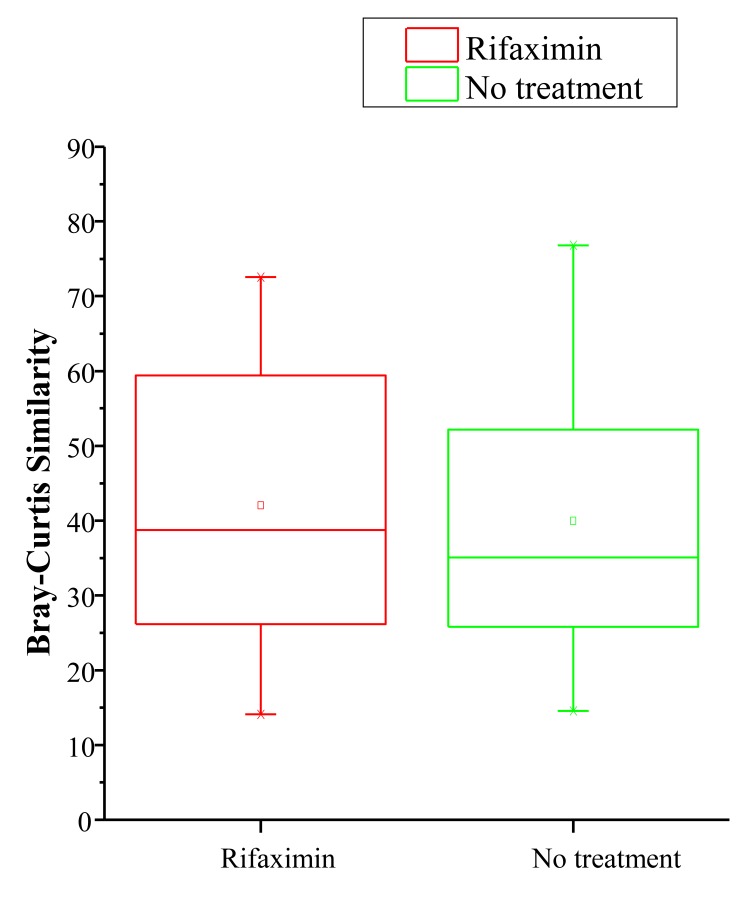
Distribution of Bray-Curtis similarity values for patients with HIV+ in either no treatment or Rifaximin-treatment arms. Shown is the distribution of Bray-Curtis similarity values for each individual from visit 1 to visit 2. Mann-Whitney test indicates that at the 0.05 level, the two arms are NOT significantly different. Bray-Curtis similarity values are based on untransformed, but standardized, taxon abundance data at the level of family.

**Supplemental Figure 2.**
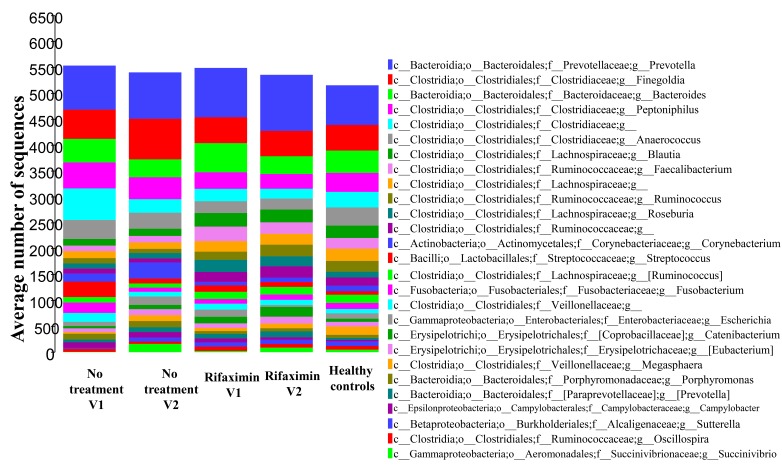
Genus-level microbial community composition of fecal microbiome samples from individuals in the Rifaximin and placebo experimental arms. A set of 21 matched healthy controls (HIV-) are also shown. Average abundance of each taxon is shown among 31 individuals (Rifaximin arm) and 17 individuals (placebo). Each sample was rarefied to 6500 sequences and average values for each taxon are shown. Taxa greater than 1% of the total dataset are shown.
